# Deep learning for early dental caries detection in bitewing radiographs

**DOI:** 10.1038/s41598-021-96368-7

**Published:** 2021-08-19

**Authors:** Shinae Lee, Sang-il Oh, Junik Jo, Sumi Kang, Yooseok Shin, Jeong-won Park

**Affiliations:** 1 Department of Conservative Dentistry, Gangnam Severance Hospital, College of Dentistry, Yonsei University, 146-92 Dogok-dong, Gangnam-gu, Seoul, 135-720 Korea; 2 SELVAS AI Inc., Seoul, 08594 Korea; 3 Department of Conservative Dentistry, College of Dentistry, Yonsei University, Seoul, Korea

**Keywords:** Dental caries, Digital radiography in dentistry, Periapical radiographs

## Abstract

The early detection of initial dental caries enables preventive treatment, and bitewing radiography is a good diagnostic tool for posterior initial caries. In medical imaging, the utilization of deep learning with convolutional neural networks (CNNs) to process various types of images has been actively researched, with promising performance. In this study, we developed a CNN model using a U-shaped deep CNN (U-Net) for caries detection on bitewing radiographs and investigated whether this model can improve clinicians’ performance. The research complied with relevant ethical regulations. In total, 304 bitewing radiographs were used to train the CNN model and 50 radiographs for performance evaluation. The diagnostic performance of the CNN model on the total test dataset was as follows: precision, 63.29%; recall, 65.02%; and F1-score, 64.14%, showing quite accurate performance. When three dentists detected caries using the results of the CNN model as reference data, the overall diagnostic performance of all three clinicians significantly improved, as shown by an increased sensitivity ratio (D1, 85.34%; D1′, 92.15%; D2, 85.86%; D2′, 93.72%; D3, 69.11%; D3′, 79.06%; *p* < 0.05). These increases were especially significant (*p* < 0.05) in the initial and moderate caries subgroups. The deep learning model may help clinicians to diagnose dental caries more accurately.

## Introduction

Dental caries, also known as tooth decay, is one of the most common chronic diseases worldwide. According to the American Dental Association, dental caries can be classified as normal, initial, moderate, or extensive according to the lesion extent^1^. The early detection of initial dental caries can prevent invasive treatment, thereby saving healthcare costs. However, detecting posterior initial proximal caries with clinical examinations alone is difficult, and bitewing radiography is helpful as the gold standard for diagnosing demineralized proximal caries^2^. The combination of bitewing radiographs and a visual inspection is a routine diagnostic approach for proximal caries detection^3^. Besides radiographs, fiber optic transillumination and fluorescence-based methods, such as DIAGNOdent (KaVo, Charlotte, NC, USA), are other ways to detect dental caries^4^. However, these methods have limitations in detecting posterior initial proximal caries^5^ and incur additional device costs. Bitewing radiograph is still the most reliable and widely used method in clinical situations.

Although radiography is recommended as a diagnostic method, the detection of dental caries using radiographs can be subjective. Major differences exist across observers in terms of whether caries lesions are detected, even using the same radiograph. Factors such as the quality of the radiograph, viewing conditions, the dentist’s expectations, variability across examiners (in particular, whether a dentist leans towards or minimizes caries diagnoses), and the length of time per examination cause discrepancies in interrater agreement^6,7^. In a previous study, 34 raters showed considerable variation when examining the same bitewing radiographs, with mean kappa values of 0.30–0.72 for the presence or absence of dental caries and the degree thereof^6,8^. A lack of consistency is a significant problem, especially for the detection of initial dental caries^9^.

In recent years, researchers have actively explored the utilization of deep learning with convolutional neural networks (CNNs) to process various types of medical images, with promising performance. The usage of deep learning for the diagnosis of diseases is increasing, and deep learning has shown precise and expeditious detection with improved clinical outcomes^10^. In dentistry, the use of deep convolutional networks has been investigated since 2015. The U-Net was employed by Ronneberger to analyze dental structure segmentation on bitewing radiographs^11^. Subsequently, multiple deep learning models for diagnosing dental caries or lesion detection on dental X-ray images have been studied^12–14^. Most extant research has been limited to analyses of the detection performance of deep learning models, and some recent papers have compared diagnostic performance between deep learning models and clinicians^9,15^. However, no study has yet investigated the changes that result from using deep learning models in clinical situations, or how clinicians can benefit from deep learning models.

In this study, we developed a U-Net CNN model^11^ for dental caries detection on bitewing radiographs through an analysis of dental structure and differences in radiographic density on radiographs without special manipulation, and investigated whether the proposed model can help clinicians diagnose dental caries in actual clinical settings. The null hypothesis tested was that there would be no difference when the clinicians referred to the results of the CNN model when diagnosing dental caries on bitewing radiographs.

## Materials and methods

### Data collection

This study was approved by the Institutional Review Board of Yonsei University Gangnam Severance Hospital and Yonsei University Dental Hospital (IRB No. 3-2019-0062 & No. 2-2019-0031) and all research was carried out in accordance with relevant guidelines and regulations. This study was a retrospective study, for which the requirement for informed consent was waived by the Institutional Review Board of Yonsei University Gangnam Severance Hospital and Yonsei University Dental Hospital due to its data source and methods. The radiographs were randomly selected by two dentists from the archive of the Department of Conservative Dentistry, Yonsei University containing bitewing radiographs taken from January 2017 to December 2018 for caries diagnosis and treatment. Radiographs including only permanent teeth were used for data, with no additional information about the patients (e.g. sex, age, or other clinical information). We tried to include a variety of cases that were as similar as possible to those encountered in real-world clinical situations. Radiographs with low image quality, excessive distortion, or severe overlapping of proximal surfaces due to the anatomical arrangement of particular teeth were excluded, because those features would interfere with a precise caries diagnosis. The bitewing radiographs were taken with the aid of a film-holding device (RINN SCP-ORA; DENTSPLY Rinn, York, PA, USA) using a dental X-ray machine (Kodak RVG 6200 Digital Radiography System with CS 2200; Carestream, Rochester, NY, USA).

**Figure 1:**
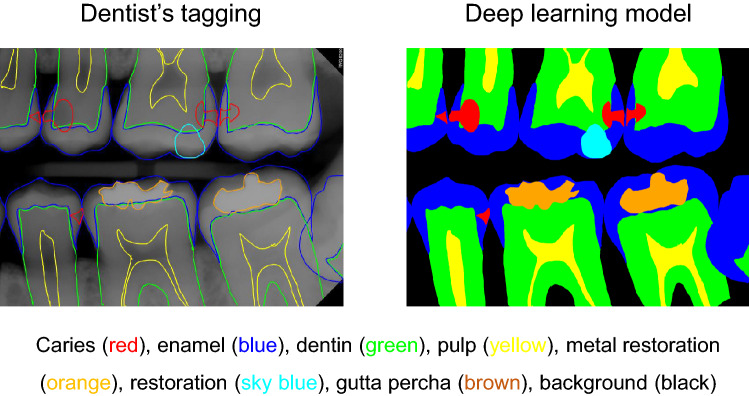
Example of the analysis of dental structures and caries tagging**.** The observers drew lines for the segmentation of dental structures (enamel, dentin, pulp, metal restoration, tooth-color restorations, gutta percha) and dental caries on the bitewing radiographs. *No software was used to generate the image. The picture used in Figure is a file printed using the source code that we implemented ourselves.

The collected data were transferred to a tablet (Samsung Galaxy Note 10.1; Samsung Electronics Co., Suwon, South Korea) as Digital Imaging and Communications in Medicine files, and the two well-trained observers (postgraduate students of the Department of Conservative Dentistry, with a minimum clinical experience of 5 years) examined the dental images sequentially. The observers were allowed to adjust the density or contrast of radiographs as they wished with no time limitation. The observers drew lines for the segmentation of dental structures (caries, enamel, dentin, pulp, metal restorations, tooth-colored restorations, gutta percha) (Fig. 1) on the bitewing radiographs. All types of dental caries (e.g., proximal, occlusal, root and secondary caries) that can be observed on bitewing radiographs were tagged regardless of the severity. Discrepancies in caries tagging (e.g., regarding the presence or absence of the caries and the size of the caries) were initially resolved by consensus between the two observers, and if the disagreement persisted, it was resolved by another author.

### Training the convolutional neural network

The bitewing radiographs were directly used as diagnostic data for CNN without specific pre-processing (e.g. image enhancement and manual setting of the region of interest). The models were trained using each bitewing radiograph with 12-bit depth, which is the manufacturer’s raw format for bitewing radiographs, and its paired binary mask, while only the radiograph itself was fed into the models to detect the target regions for testing. The radiographs were scaled to the size of $572 \times 572$ to be used as input for the networks. In total, 304 bitewing radiographs, which were randomly divided into two groups (149 radiographs for ${\mathcal{D}}_{{\text{A}}}$, which was used for training on both structure and caries segmentation, and 105 radiographs for ${\mathcal{D}}_{B}$, which was used for training on caries segmentation alone, as described below in greater depth) and a group of 50 radiographs with no dental caries (${\mathcal{D}}_{{\text{C}}} )$, were used to train the deep learning model, while 50 radiographs (${\mathcal{D}}_{{\text{D}}} )$ were used for performance evaluation. To evaluate the generality of the trained models without dataset selection biases, cross-validation was applied to the datasets ${\mathcal{D}}_{{\text{A}}} ,\;{\mathcal{D}}_{B}$, and ${\mathcal{D}}_{{\text{C}}}$. Each dataset was randomly split into six different folds exclusive of each other. The six folds were grouped into training, validation, and test sets at a 4:1:1 ratio for each repeat, with six rounds of repeated fold shuffling. In other words, each fold was not just a training set for a cross-validation model, but also a test set for the other cross-validation model. During the training time, the optimal model epoch was selected by evaluating the error rate of the radiographs of the validation set. After finishing the training, the test set, which was a totally independent fold from both the training and validation sets, was used to measure the final performance by using the model with the optimal epoch. If there had been pre-existing criteria for grading the level of difficulty of caries detection from the radiographs, we would have deliberately split the entire dataset into training, validation, and test sets according to those criteria. However, there are no standard criteria that could be used to gather each set without difficulty bias, which refers to the possibility that all the hard cases from the collected data could be included in the training set, whereas the easiest cases would be in the test set. Therefore, training and evaluating the model by randomly organized multiple cross-validation folds reduces the performance gaps between the test set and real-world data caused by dataset selection bias. Two augmentation processes were applied to the training set to train the model. Image augmentation, including intensity variation, random flipping, rotation, elastic transformation, width scaling, and zooming, was equally applied first to each radiograph and its paired binary mask. The image augmentation process acts as a regularizer that minimizes overfitting by randomly incorporating various image artifacts into the image dataset. Subsequently, mask augmentation consisting of random kernel dilations and elastic transformation was applied to only the binary masks. Mask augmentation was designed to reflect label inconsistencies among dentists. To detect the caries and structure regions from each input radiograph, this study used the U-Net architecture, a famous architecture for segmenting target regions on the pixel level. The U-Net architecture includes a convolutional part and an up-convolutional part. The convolutional part has the typical structure of convolutional neural networks with five convolutional layers. Contrastively, the up-convolutional part performs an up-sampling of the feature map by taking a concatenated output of the previous layer and the opposite convolutional layer as an input. Then, a $1 \times 1$ convolution maps the dimensionality of the feature maps to the desired number of classes. Finally, a softmax layer outputs a probability map where each pixel indicates the probability for each class within a range of $\left[ {0, 1} \right]$ [ref. Softmax]. The network was optimized by the adaptive moment estimation optimizer with an initial learning rate of 0.00001 [ref. Adam optimizer]. Details on model architecture are provided in the Supplementary Fig. S1 online.

To accurately detect caries regions from the input bitewing radiographs, we designed a two-step process: detection and refinement. To do this, the U-Net was trained on two models: the U-Net for caries segmentation (U-CS), and the U-Net for structure segmentation (U-SS). The purpose of the U-CS is to extract carious segments from the input radiograph, whereas the U-SS segments dental structures. The input radiographs and their corresponding binary masks were used to train two U-Nets. The target regions for the U-CS and the U-SS were carious regions and structures, respectively. To train U-SS, the ${\mathcal{D}}_{{\text{A}}}$ dataset was used and showed quite accurate performance in segmentation of dental structures with a small training dataset. In contrast, the U-CS was trained by consecutively accumulating the datasets from ${\mathcal{D}}_{{\text{A}}}$ to ${\mathcal{D}}_{C}$ to observe the change in performance according to the features of the training dataset and the number of radiographs. Additionally, we introduced a penalty loss to overcome the limitation from the small scale of the training dataset, which can give rise to a large number of false detections. The penalty loss term was designed to assign a penalty to predicted carious regions on an input radiograph having no actual carious regions during training.

**Figure 2:**
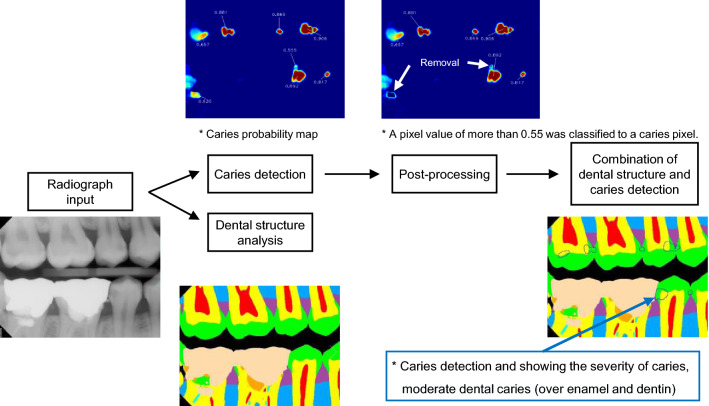
Flowchart of the detection of dental caries in the deep learning model, showing two models: the U-Net for caries segmentation (U-CS), and the U-Net for structure segmentation (U-SS). *No software was used to generate the image. The picture used in Figure is a file printed using the source code that we implemented ourselves.

By simultaneously feeding each input radiograph into the U-SS and the U-CS, a caries probability map and a structure probability map were generated. The program was constructed to visualize detected dental caries as an area on the bitewing radiograph and also to show the degree of dental caries numerically. For the caries probability map, a pixel value of more than 0.55 was classified as a caries pixel. To find the threshold value of 0.55, we iteratively observed the agreement between the outputs of the trained model and experts’ diagnoses for the validation sets, while changing the threshold value from 0.01 to 0.99 with a step size of 0.01. In this process, the threshold value of 0.55 showed the optimal results in terms of agreement. To reduce the likelihood of false detection, areas of caries detected by the U-CS without overlap with enamel or dentin regions were eliminated. Figure 2 shows an overview of caries detection and false detection refinement.

### Performance evaluation of the CNN model

To evaluate the performance of dental caries detection, the assessments were computed at the caries component level. If a blob classified as caries overlapped with a ground truth caries region at a specific ratio, we regarded the blob as a hit. We measured the precision (also known as positive predictive value (PPV), $TP/\left( {TP + FP} \right)$, %), recall (also known as sensitivity, $TP/\left( {TP + FN} \right)$, %), and F1-score (${\text{F}}1\;{\text{score}} = {{\left( {2\left( {precision*recall} \right)} \right)} \mathord{\left/ {\vphantom {{\left( {2\left( {precision*recall} \right)} \right)} {\left( {precision + recall} \right)}}} \right. \kern-\nulldelimiterspace} {\left( {precision + recall} \right)}}$) according to the overlap ratio ($\theta$), which was set to 0.1, between the agreed-upon and predicted carious regions. We computed the final result by summing the number of caries from the test set of the cross-validation models on each dataset ${\mathcal{D}}_{{\text{A}}} ,{ }{\mathcal{D}}_{{\text{B}}} ,$ and ${\mathcal{D}}_{{\text{C}}}$, whereas ${\mathcal{D}}_{{\text{D}}}$ was not included.

### Performance evaluation and comparisons of dentists (before vs. after revision) with assistance of the CNN model

To assess whether the developed deep learning model can support the diagnosis of dentists, we used ${\mathcal{D}}_{{\text{D}}}$, the evaluation dataset containing 50 radiographs. Specifically, we explored how dentists’ diagnoses changed before and after they referred to the predictions of dental caries made by the deep learning model. To do this, the deep learning model, which was previously trained with 304 training images, was used to detect dental caries in 50 radiographs, and three dentists (working in clinics with a clinical experience of 4–6 years) were instructed to tag dental caries independently, without any consultations. A few weeks later, the three dentists diagnosed the same radiographs, but with two distinguishable guidance lines, comprising the regions of dental caries predicted by the model and their previous diagnosis. The three dentists were instructed to revise (modify, delete, or add) their previously tagged dental caries by referencing the dental caries results detected by the deep learning model. As a result, each radiograph had seven sets of dental caries regions in total: six from dentists, and one from the model. To evaluate the validity of the model as a diagnostic support system, we analyzed the changes between first and second diagnoses of each dentist by measuring the PPV (%), sensitivity(%) and F1-score.Three dentists reached consensus on dental caries detection in ${ }{\mathcal{D}}_{{\text{D}}}$, the evaluation dataset containing 50 radiographs, and divided the agreed-upon dental caries into 3 subgroups according to the severity of caries (initial, moderate, and extensive). The radiographic presentation of the proximal caries was classified as follows: sound: no radiolucency; initial: radiolucency may extend to the dentinoenamel junction or outer one-third of the dentin; moderate: radiolucency extends into the middle one-third of the dentin; and extensive: radiolucency extends into the inner one-third of the dentin^1^. If the same area was included, the same dental caries was considered to have been detected regardless of the overlap ratio on the caries lesion level; using this criterion, the sensitivity (%) was calculated according to the severity of the agreed-upon dental caries.

### Statistical analysis

The diagnostic performance for readers and the U-Net CNN model was calculated in terms of the PPV (%), sensitivity (%), and F1-score. To compare the PPV and sensitivity between readers and the U-Net CNN model, generalized estimating equations (GEEs) were used, while the F1-score was compared using the bootstrapping method (resampling: 1000). All statistical analyses were performed using SAS (version 9.4, SAS Inc., Cary, NC, USA) and R package (version 4.1.0, http://www.R-project.org). The significance level was set at alpha = 0.05.

## Results

### Diagnostic performance of the CNN model

The diagnostic performance of the final CNN model on the total test dataset (${\mathcal{D}}_{{{\text{A}},{\text{B}},{\text{C}}}}$) was as follows: precision, 63.29%; recall, 65.02%; and F1-score, 64.14%.

The deep learning program was quite accurate and showed a stable pattern of dental caries detection performance. All types of dental caries (root caries, secondary dental caries, and gaps under restoration) recognizable on bitewing radiographs, other than proximal dental caries, were detectable. However, the false detection rate of dental caries was somewhat higher when the quality of the radiographs was low, dental overlap was severe, and when the bitewing images included the third molar.

### Diagnostic performance of dentists (before vs. after revision) with assistance of the CNN model

The PPV, sensitivity, and F1-score between dentists (before vs. after revision) and the model are shown in Table 1. When the three dentists detected dental caries with support from the deep learning model, their sensitivity increased, while the PPV decreased. Statistically significant differences (*p* < 0.05) were found in the overall sensitivity and PPV of the dentists (before vs. after revision). However, there was no significant difference (*p* > 0.05) in the F1 score of the dentists (before vs. after revision). When the three observers independently diagnosed dental caries on 50 images, the number of dental caries tagged by each was as follows: D1, 177; D2, 182; and D3, 155. After revision of their diagnoses referencing the dental caries detection results of the deep learning model, the final number of dental caries tagged by the three observers increased as follows: D1, 211; D2, 202; D3, 175. When the clinicians detected dental caries with reference to the results of the CNN model, they detected more caries.

**Table 1:** Diagnostic performance of three dentists (before vs. after revision) and the CNN model on ${\mathcal{D}}_{{\text{D}}}$, the evaluation dataset (subgrouped according to the severity of the agreed-upon dental caries).

	Overall			Initial	Moderate	Extensive
	Sensitivity (95% CI)	PPV (95% CI)	F1 score (95% CI)	Sensitivity (95% CI)	Sensitivity (95% CI)	Sensitivity (95% CI)
**Before revision**						
Dentist 1 (1)	85.34(80.32–90.36)^†^	100(100–100)^†^	92.07(88.95–94.77)	77.89(69.54–86.24)^†^	90.54(83.87–97.21)^†^	100(100–100)
Dentist 2 (2)	85.86(80.92–90.80)^§^	100(100–100)^§^	92.42(89.28–95.06)	80(71.96–88.04)^§^	91.89(85.67–98.11)^§^	90.91(78.9–100)
Dentist 3 (3)	69.11(62.56–75.66)*	100(100–100)*	81.72(77.17–85.98)	56.84(46.88–66.8)*	78.38(69–87.76)*	90.91(78.9–100)
CNN model (4)	83.25(77.95–88.55)	76.08(70.30–81.86)	79.46(74.81–83.29)	74.74(66–83.48)	90.54(83.87–97.21)	95.45(86.74–100)
**After revision**						
Dentist 1 (5)	92.15(88.34–95.96)^†^	89.34(85.03–93.65)^†^	90.67(87.53–93.73)	86.32(79.41–93.23)^†^	97.3(93.61–100)^†^	100(100–100)
Dentist 2 (6)	93.72(90.28–97.16)^§^	94.21(90.89–97.53)^§^	94(91.4–96.14)	90.53(84.64–96.42)^§^	97.3(93.61–100)^§^	95.45(86.74–100)
Dentist 3 (7)	79.06(73.29–84.83)*	96.79(94.02–99.56)*	87.02(82.99–90.2)	66.32(56.82–75.82)*	89.19(82.12–96.26)*	100(100–100)

Statistical analyses were performed using SAS (version 9.4, SAS Inc., Cary, NC, USA), GEEs were used.

*CNN* Convolutional neural network, *CI* Confidence intervals.

^†^Difference between Dentist 1 (before revision, (1)) vs. Dentist 1 (after revision, (5)), *p* < 0.05.

^§^Difference between Dentist 2 (before revision, (2)) vs. Dentist 2 (after revision, (6)), *p* < 0.05.

*Difference between Dentist 3 (before revision, (3)) vs. Dentist 3 (after revision, (7)), *p* < 0.05.

**Figure 3:**
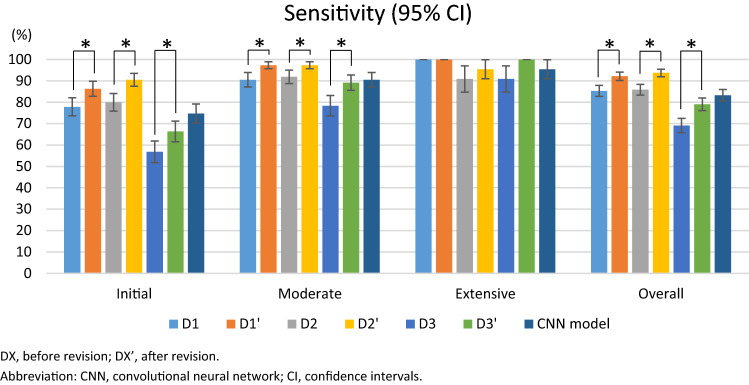
Comparison of diagnostic performance (sensitivity) between three dentists (before and after revision) and the deep learning model. Mean ± standard deviation, CNN convolutional neural network, CI confidence intervals. *The significance level was set at alpha = 0.05 in the post hoc analysis. Statistical analyses were performed using SAS (version 9.4, SAS Inc., Cary, NC, USA), GEEs were used.

When the results were analyzed according to the severity of the dental caries, the clinicians tended to be less accurate in detecting initial caries lesions with lower sensitivity (Fig. 3). When the three clinicians referred to the deep learning results as a second opinion, the sensitivity of all three clinicians for initial and moderate caries, which are easy to miss, significantly improved (*p* < 0.05).

## Discussion

In this study, we developed a CNN model using U-Net for dental caries detection on bitewing radiographs with quite accurate detection performance and confirmed that this model could meaningfully help clinicians detect caries in clinical situations.

In the present study, when three clinicians performed dental caries tagging on the evaluation dataset, the lowest sensitivity ratio was found for initial caries, followed in ascending order by moderate and extensive caries. This result coincides with the study of Cantu-Garcia^9^. Clinicians were more likely to miss initial caries on bitewing radiography and showed lower accuracy than for extensive caries. However, when clinicians referred to the results of the deep learning model when making diagnoses, the sensitivity ratio increased in every group of caries severity. This change was particularly remarkable in the initial and moderate groups, with statistically significant differences (*p* < 0.05). Therefore, guidance from the deep learning model was meaningful as a way to help clinicians detect early caries that could otherwise be mistakenly missed.

In this study, after revision, the three dentists’ sensitivity significantly increased, while the PPV significantly decreased (*p* < 0.05); accordingly, the null hypothesis was rejected. Furthermore, after revision, the number of caries tagged by all three observers increased. When clinicians received assistance from the CNN model, they diagnosed more dental caries accurately with increased sensitivity; however, over-detection of caries increased the false-positive rate, resulting in a lower PPV. When the model results were referenced, it was more common for the observer to detect additional dental caries or to increase the extent of caries than it was for the observer to remove the caries or reduce the affected area. Thus, referencing deep learning results as a second opinion was helpful for finding dental caries that had not been discovered initially due to time restraints or mistakes. Since the U-Net CNN model selects candidate carious areas that can be missed by mistake in a busy clinical situation, it can prompt clinicians to look at the relevant areas once more, thereby reducing the likelihood of missing the appropriate timing for treatment as a result of not catching dental caries early.

Thus, it may be helpful for clinicians to reference the dental caries detection results of a deep learning model as a second opinion when using bitewing radiography for caries detection, as a way to improve diagnostic accuracy and not to miss the opportunity to provide preventive treatments, especially for early dental caries lesions. It should be kept in mind that bitewing radiography, as used in this study, is vulnerable to false-positive and false-negative diagnoses^16^, and therefore may not be sufficient for diagnosing dental caries in images where proximal surfaces overlap or distortion is severe. In this study, clinicians often disagreed about the presence or absence of initial caries in radiographs with overlapping proximal surfaces, and the CNN model also showed more false-positive errors in detecting caries in such regions. When there are differences in radiographic density due to a tooth fracture or other enamel defects, not dental caries, the CNN model tended to show false-positive responses. In contrast, clinicians make a final decision on the presence of caries through logical thinking based on their knowledge of the shape or pattern of dental caries progression. This is thought to be an inevitable limitation of bitewing radiography, and in such cases, the presence of caries would eventually have to be determined by a visual or clinical examination using the information obtained from radiography. When an impacted third molar overlaps with the second molar, the CNN model we trained tended to show false-positive responses because of the lack of training data for cases of superposition with other unexpected features; this limitation should be overcome by increasing the size of the training dataset. However, since such areas can be excluded as false-positive errors by clinicians, they are not thought to pose a major problem in clinical settings.

The deep learning model developed in this study showed reliable dental caries detection performance as a result of training with 304 images, which included 763 dental caries. However, a limitation is that the training data set was small. Therefore, two data augmentation processes were performed to compensate for this limitation. Furthermore, only results that equaled or exceeded the threshold value (size of dental caries, prediction probability: 0.55) were considered to be detected dental caries, which was appropriate for reducing false detection results and improving detection performance. Additionally, the model was trained using 50 images with no dental caries (dataset D_C_), and a penalty for false detection was applied to the CNN model to reduce false positives. Subsequently, improvements in dental caries detection performance were confirmed. Furthermore, to avoid false positives, caries was detected from only the enamel and dentin areas by combining the results of caries detection (U-CS) and tooth segmentation (U-SS).

In future research, the ongoing addition of training data will be needed, which will improve the accuracy of detection of the CNN model. Additionally, to obtain meaningfully high levels of accuracy in a clinical setting, it is necessary to check whether the same dental caries detection performance is shown when using bitewing radiography in primary teeth or radiography obtained by devices at multiple institutions, instead of only using bitewing radiography obtained at a single institution This study used bitewing radiographs obtained at two hospitals (Gangnam Severance and Yonsei University Dental Hospital). The X-ray equipment used at both institutions was identical, and both sets of data showed a similar level of performance in dental caries detection.

In this study, the presence of dental caries was evaluated only using radiographs, without visual or clinical examination data. Therefore, this study used the data of agreed-upon dental caries based on the consensus of the observers, which could be biased toward clinicians’ prejudices, and has the limitation of lacking gold-standard findings such as histological assessment or a visual-tactile assessment with proximal tooth separation that would conclusively demonstrate the actual presence of dental caries. Therefore, clinicians' diagnostic accuracy was likely to have been higher in our study than in other research. Nonetheless, for future research, it would be preferable to design a prospective study using methods such as visual-tactile assessment as the gold standard for verifying the actual presence of dental caries in the analysis of clinicians’ diagnostic performance before and after revision with assistance of the CNN model.

Regardless of how much the CNN model's diagnostic performance improves, there are bound to be occasional false-positive errors. Furthermore, in this study, clinicians unintentionally found too many dental caries when they referred to the results of the CNN model, resulting in an increased false-positive rate and a decreased PPV. The problem that would be expected if the CNN model is used to diagnose dental caries in clinical settings is that the false-positive results of the deep learning model would be grounds for unnecessary treatment or overtreatment. Clinicians should not wholly rely on artificial intelligence-based dental caries detection results, but should instead use them only for reference. Through additional clinical examinations and an assessment of patients’ systemic state, overall oral condition, and overall caries risk, clinicians should determine the final diagnosis and treatment plan on their own initiative.

## Conclusion

The findings of the present study revealed that referencing the dental caries detection results of a deep learning model as a second opinion may help clinicians to diagnose early dental caries more accurately. However, the addition of more training data is needed to achieve more stable and precise results.

## Supplementary Information

Supplementary Information.

## Data Availability

All data are included in this published article.
