# Supplementary material for: Deep learning for early dental caries detection in bitewing radiographs
Source: Sci Rep. 2021 Aug 19;11:16807. doi: 10.1038/s41598-021-96368-7 (PMC8376948; doi:10.1038/s41598-021-96368-7)
Supplement: Supplementary file 1 — Supplementary Information. [file 41598_2021_96368_MOESM1_ESM.docx]

**Details of architecture**

The U-Net architecture includes a convolutional part and an up-convolutional part (Fig. A1). In convolutional part including five layers, each layer consist of two $3\times3$ convolutions followed by a rectified linear unit (ReLU) function. For the first four layers, a $2\times2$ max pooling with stride 2 is assigned to the output of the ReLU. By passing through each layer of convolutional part, the number of feature channels is doubled. The four deconvolutional layers are subsequent to the last layer of the convolutional part. The deconvolutional layers take the both outputs of the previous layer and the opposite layer as an input by concatenating them.

Fig. S1. The U-net architecture of the model.
